# Adult-Onset Multisystem Langerhans Cell Histiocytosis With Bone and Hepatic Involvement: A Rare Case With Radiologic-Pathologic Correlation

**DOI:** 10.7759/cureus.86919

**Published:** 2025-06-28

**Authors:** Yanni Zulia, Christopher D Louviere, Abdullah Mohamed, Anwer Siddiqi, Renato Abu Hana

**Affiliations:** 1 Radiology, University of Florida College of Medicine - Jacksonville, Jacksonville, USA; 2 Pathology and Laboratory Medicine, University of Florida College of Medicine - Jacksonville, Jacksonville, USA; 3 Pathology, University of Florida College of Medicine - Jacksonville, Jacksonville, USA

**Keywords:** ct angiogram, immunostaining, langerhans cell histiocytosis(lch), metastasis, reticuloendothelial system

## Abstract

Langerhans cell histiocytosis (LCH) is a rare proliferative disorder of histiocytic dendritic cells that can mimic metastatic malignancies due to its potential for multiorgan involvement. Histopathologic confirmation is essential for accurate diagnosis. As such, biopsy is imperative, especially in patients who present with hepatic lesions. Adjunctive liver imaging can help confirm the diagnosis, but is rarely performed. Little information is available on imaging features of hepatic involvement in LCH. Here we report a case of a 22-year-old man with a fracture of his cervical spine suspicious for pathological fracture. However, liver imaging and confirmatory histopathology revealed LCH instead of metastatic spread.

## Introduction

Adult-onset Langerhans cell histiocytosis (LCH) is an exceptionally rare proliferative disorder involving histiocytic dendritic cells that can affect multiple organ systems. The condition typically affects pediatric populations, with the average age of adult-onset LCH being between 30 and 40 years. Histiocytic dendritic cells characteristically express CD1a, S100, and CD207 (Langerin) surface antigens and contain distinctive intracytoplasmic organelles known as Birbeck granules, which aid in differentiating LCH from other histiocytic disorders [1].

LCH can be categorized based on the number of lesions and the extent of organ system involvement. The unifocal form typically affects a single site, such as bone or lung. The multifocal unisystem form involves multiple lesions within a single system, often the skeletal or reticuloendothelial system (including liver, spleen, lymph nodes, and skin). The most severe, the multifocal multisystem form, presents with disseminated disease affecting multiple organ systems, particularly within the reticuloendothelial system [2-4].

As LCH involves the reticuloendothelial system, liver involvement, in particular, can vary between adult and pediatric populations. In cases of hepatic LCH, biopsy with staining for characteristic immunomarkers remains the cornerstone of diagnosis and disease staging. However, accompanying imaging can play a crucial diagnostic role. Unfortunately, due to the rarity of the disease, especially the multifocal form in adults, there are few documented cases with detailed liver imaging findings.

In this report, we present an adult-onset case of a pathological cervical spine fracture, initially concerning for metastatic disease, in which the diagnosis of LCH was guided by imaging and confirmed with liver biopsy.

## Case presentation

**Figure 1:**
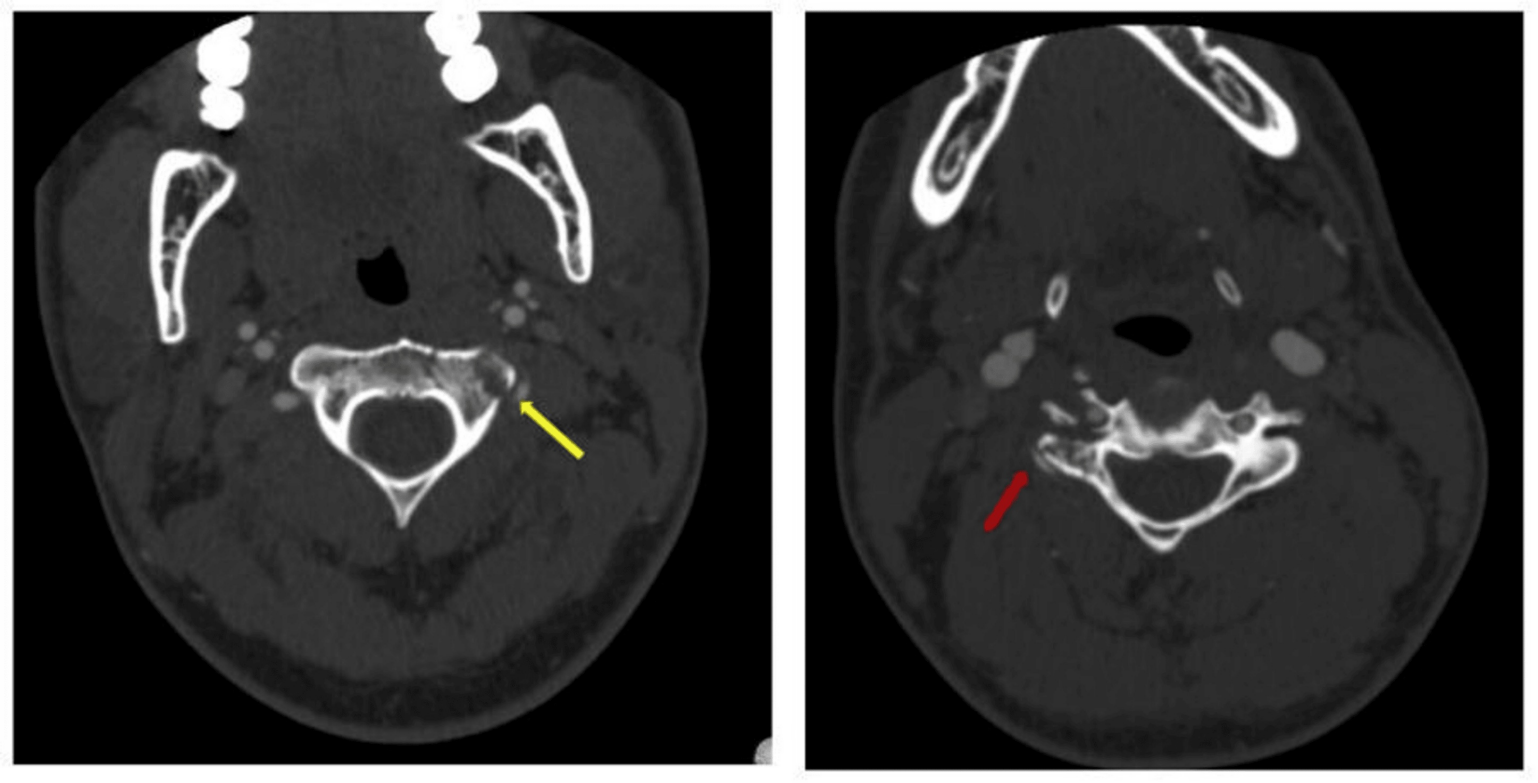
CTA neck axial showing nontraumatic displaced fractures of the left transverse process of C2 (yellow arrow) and right transverse process of C4 (red arrow), concerning for pathological fracture. CTA, computed tomographic angiography.

A 22-year-old male with no significant past medical history presented to the emergency department after experiencing progressive, non-traumatic, and severe neck pain over the past three weeks that had failed conservative therapy. A CT angiogram of his head and neck was obtained, which showed a displaced fracture of the left transverse process of C2 and right transverse process of C4, extending into the vertebral foramen. Given this atypical presentation in the absence of trauma, there was concern for an underlying malignant process. MRI of the cervical spine was obtained, which demonstrated lytic lesions at the left transverse process of C2 and the right body, pedicle, and lateral mass of C4 (Figure 1).

An oncology consultation was obtained, and a CT of the chest, abdomen, and pelvis with contrast was performed as part of the workup to identify a potential primary tumor and assess for metastatic disease. Imaging revealed multiple well-defined, rounded hypodense lesions in the liver, raising significant concern for metastases. However, no primary tumor was identified.

**Figure 2:**
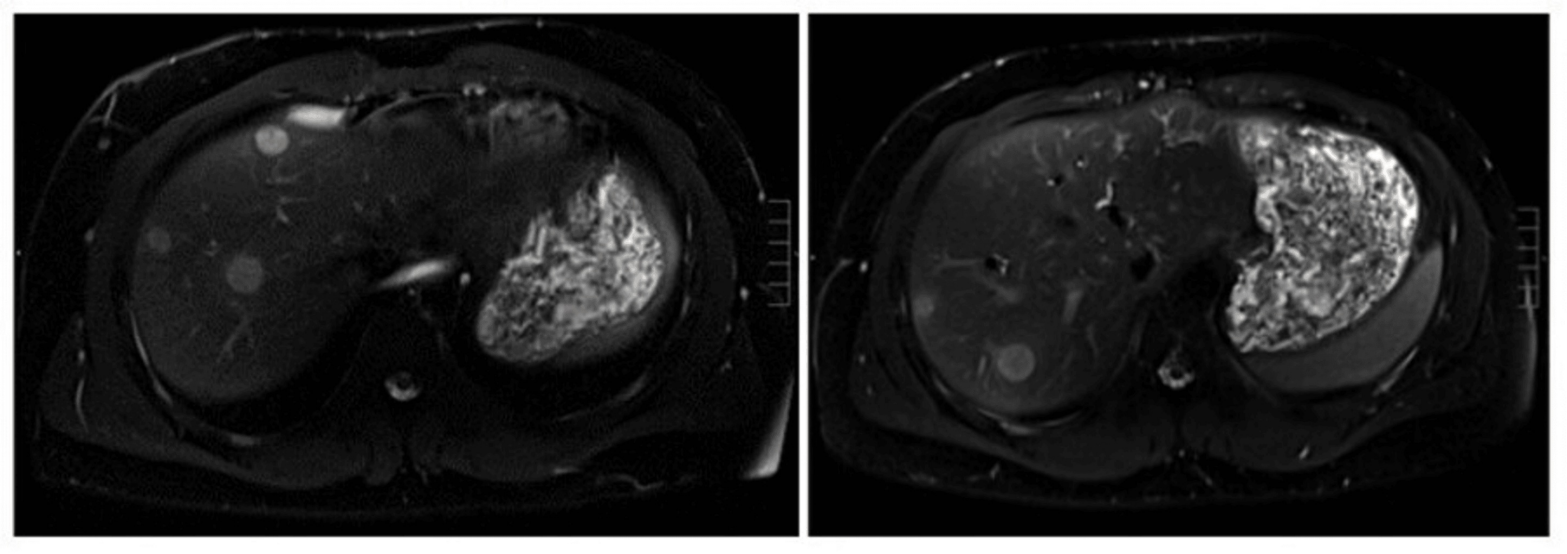
MRI abdomen T2 sequence showing multiple well-defined, rounded hyperintense liver lesions, concerning for metastatic disease. MRI, magnetic resonance imaging.

An MRI of the liver was obtained to evaluate for infectious etiologies. It revealed multiple well-defined, rounded hepatic lesions, the largest measuring up to 1.6 cm, demonstrating restricted diffusion, heterogeneous contrast enhancement, and peripheral edema, findings once again concerning for metastatic disease (Figure 2).

**Figure 3:**
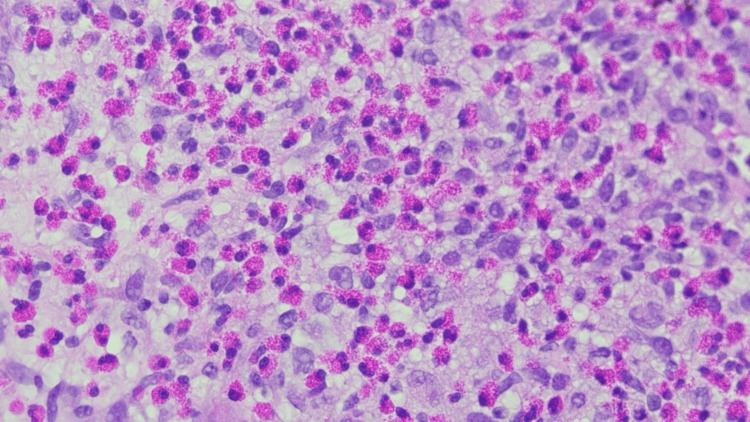
Liver biopsy showing Langerhans cells with nuclear membrane irregularity and admixed eosinophils. H&E stain at (40×). H&E, hematoxylin and eosin.

**Figure 4:**
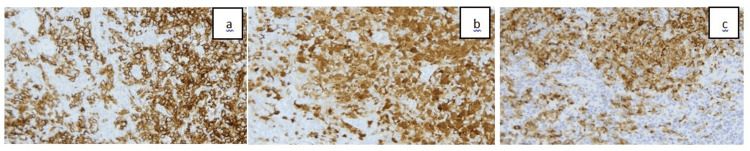
Tumor cells are positive for immunostains: (a) S-100, (b) CD1a, and (c) Langerin at (20×).

Given the uncertain diagnosis, a liver biopsy was subsequently performed. Histopathology demonstrated a mixed histiocytic and eosinophilic infiltrate with immunostaining positive for S-100, CD1a, CD68, CD45, and Cyclin D1. Langerin immunostaining was also positive, supporting the diagnosis of LCH (Figures 3, 4).

After discharge, the patient was advised to follow up with Hematology-Oncology for further medical management. However, there is no documentation of this follow-up visit to date. He is currently being managed by his primary care provider, who is treating his symptoms with opioids and has initiated a referral to pain management.

## Discussion

In this case, LCH presented as a pathological cervical fracture on MRI, which demonstrated enhancing spinal lesions at the fracture site and cervical vertebra. Interestingly, aside from back pain secondary to pathologic fracture, the patient did not report any additional signs and symptoms common among LCH patients, such as jaundice, pruritus, fever, or weight loss.

Bone lesions are the most common radiographic manifestation of LCH, with the skull, particularly the calvarium, being the most frequently affected site. Other commonly involved bones include the mandible, ribs, pelvis, and long bones such as the femur. Spinal involvement is less frequent, and, when present, typically affects the thoracic and lumbar regions. Cervical spine involvement, as seen in this case, is rare and contributes to the uniqueness of the presentation [1,5,6]. Spinal involvement in this case showed MRI signaling that was consistent with other literature findings, including T1-weighted (T1W) hypointensity, T2-weighted (T2W) hyperintensity, and enhancement [1]. Progression of LCH spinal lesions may cause uniform collapse of the vertebral body, termed “vertebra plana,” which is pathognomonic, but nonspecific, for LCH [4,7]. Vertebra plana may be seen in conditions such as metastatic blood cancers, spondylitis, and bone cysts [4,7].

This case presented with liver lesions on both CT and MRI. CT demonstrated a well-defined, rounded hypodense lesion with hypo-attenuated halos, and MRI showed diffusion restriction, heterogeneous enhancement, and peripheral edema. Literature describing imaging findings in hepatic LCH is scarce, but generally reports imaging phenotypes being caused by histiocytic infiltration and biliary tree sclerosis [8].

Hao et al. describe three basic imaging phenotypes in hepatic LCH. The scattered lesion type is characterized by several focal lesions in the liver parenchyma randomly distributed without periportal abnormality [9]. The disseminated lesion type is characterized by multiple patchy lesions diffusely distributed throughout the parenchyma and portal tracts [9]. The central periportal lesion type is characterized by abnormal periportal signal intensity without any liver parenchymal involvement [9]. Our case is most consistent with the scattered lesion phenotype, as the distribution of lesions is randomly appearing without periportal involvement.

Imaging alone remains insufficient for definitive LCH diagnosis. While radiographic findings can suggest a diagnosis, findings, especially in the spine and liver, can overlap with other disease processes. Definitive diagnosis of LCH can only be established by positive immunohistochemical staining of Langerhans cells for CD1a and S100 antigens [10]. Subsequent studies have identified distinct histological stages of hepatic involvement in LCH, which include proliferative, granulomatous, xanthomatous, and fibrous phases [9,11].

This case contributes to the limited imaging literature on hepatic involvement in LCH, highlighting characteristic, yet often nonspecific, features, and presents a rare instance of cervical spine involvement. Notably, it occurred in a young adult, whereas LCH more commonly presents in the pediatric population. This underscores the importance of including LCH in the differential diagnosis of atypical hepatic imaging findings, even in adult patients. Further studies are needed to better define the radiologic spectrum of this rare entity and to promote more consistent recognition across imaging modalities.

## Conclusions

This rare case of LCH in a young adult, presenting as a pathological cervical spine fracture with hepatic lesions, underscores the importance of including LCH in the differential diagnosis of metastatic-appearing disease, particularly in the absence of a known primary neoplasm. Histopathological confirmation remains essential to establish an accurate diagnosis and to exclude other etiologies, especially metastatic cancer. This case significantly contributes to the small body of literature available on adult-onset, hepatic LCH. 
